# The Pediatric Acute Leukemia Fusion Oncogene ETO2-GLIS2 Increases Self-Renewal and Alters Differentiation in a Human Induced Pluripotent Stem Cells-Derived Model

**DOI:** 10.1097/HS9.0000000000000319

**Published:** 2019-12-16

**Authors:** Salvatore Nicola Bertuccio, Fabien Boudia, Marie Cambot, Cécile K. Lopez, Larissa Lordier, Alessandro Donada, Elie Robert, Cécile Thirant, Zakia Aid, Salvatore Serravalle, Annalisa Astolfi, Valentina Indio, Franco Locatelli, Andrea Pession, William Vainchenker, Riccardo Masetti, Hana Raslova, Thomas Mercher

**Affiliations:** 1 Department of Pediatrics, “Lalla Seràgnoli”, Hematology-Oncology Unit, Sant’Orsola-Malpighi Hospital, University of Bologna, Via Massarenti 11, 40137, Bologna, Italy.; 2 INSERM U1170, Gustave Roussy, Villejuif, France; 3 Université Paris Diderot, Paris, France; 4 Institut National de Transfusion Sanguine INTS, Paris, France; 5 Gustave Roussy, IPSC platform, Villejuif, France; 6 “Giorgio Prodi” Cancer Research Center, University of Bologna, Bologna and Department of Biomedical and Specialty Surgical Sciences, University of Ferrara, Ferrara, Italy; 7 Department of Pediatrics, Sapienza, University of Rome, Italy; 8 Université Paris-Sud, Orsay, France; 9 Equipe Labellisée Ligue Contre le Cancer, France.

The ETO2-GLIS2 fusion is associated with young and aggressive pediatric acute megakaryoblastic leukemia (AMKL) and is thought to arise *in utero*. To investigate ETO2-GLIS2 consequences in human hematopoietic cells and overcome the difficulty to access appropriate fetal stages, we engineered induced pluripotent stem cells (iPSC) to obtain hematopoietic-specific ETO2-GLIS2 expression and analyzed *in vitro* hematopoietic differentiation of IPSC toward the megakaryocytic lineage, which presents common features with human hematopoiesis during early development. As compared to controls, ETO2-GLIS2 expression induced an increased proportion of CD41^+^42^+^ megakaryocytes at several differentiation timepoints and generated a CD41^low^42^low^ population that is absent in controls. In addition, ETO2-GLIS2 enhanced proliferation and self-renewal capacities in methylcellulose assays. To understand the molecular consequences of ETO2-GLIS2 expression in human progenitors, we next performed RNA sequencing on flow purified CD41^+^42^+^ megakaryocytes and the abnormal CD41^low^42^low^ population. Compared to wild-type CD41^+^42^+^ profiles, ETO2-GLIS2-expressing CD41^+^42^+^ profiles were enriched for several ETO2-GLIS2 AMKL patients blasts signatures and an ETO2-GLIS2-dependent enhancer-associated genes signature. Importantly, these signatures were even more enriched in the ETO2-GLIS2-expressing CD41^low^42^low^ suggesting that this abnormal population more closely mimic the leukemic blasts found in patients. Of note, expression of *ERG* and *GATA1*, two master hematopoietic transcription factors, were not deregulated to the same extent as in patients’ blasts. Together, this human ETO2-GLIS2 iPSC model recapitulates differentiation alterations, increased self-renewal and transcriptional signatures observed in human AMKL and should therefore represent an interesting platform to perform future molecular and preclinical investigations.

De novo pediatric acute megakaryoblastic leukemia (AMKL) is characterized by fusion oncogenes.^1,2^ Murine transgenic models have been reported for some fusions, including OTT-MAL and MN1-FLI1.^3,4^ The *CBFA2T3-GLIS2 (ETO2-GLIS2*) fusion, present in 20% to 30% of de novo pediatric AMKL, is associated with a low number of additional genetic alterations and a dismal prognosis.^2,5–8^ It induces a deregulation in the balance between hematopoietic master regulators, including a higher expression of ERG and GATA3 associated with a drastic reduction in GATA1 activity in human AMKL cells.^9^ However, the consequences of ETO2-GLIS2 expression in normal human hematopoietic cells and its leukemogenic potential is unknown to date. In several instances, *de novo* AMKL patients are diagnosed few weeks/months after birth and in twins,^10^ suggesting that the fusions were generated in a hematopoietic stem or progenitor cell during early fetal development. Based on the observation that normal megakaryocyte differentiation from human embryonic stem cells resemble early ontogenic stages,^11^ we reasoned that hematopoietic differentiation derived from human induced pluripotent stem cells (iPSC) represents a developmentally-relevant context to model pediatric-specific AMKL fusion oncogenes.

To this aim, we used normal iPSC derived from healthy hematopoietic progenitors^12^ to perform a targeted knock-in at the AAVS1 locus, through zinc-finger nucleases-mediated recombination, to introduce a GFP-tagged *ETO2-GLIS2* fusion gene under the transcriptional control of the pan-hematopoietic human CD43 promoter (Fig. 1A). Importantly, this allows to assess ETO2-GLIS2 function independently of iPSC line-to-line variances. We identified 23 clones with homozygous integration (Supplemental Fig. 1A and supplemental Table 1, Supplemental Digital Content 1 and 2). Two clones were selected for further studies, validated for a stable karyotype (Supplemental Fig. 1B, Supplemental Digital Content 1) and pluripotency (Supplemental Fig. 1C,D, Supplemental Digital Content 1) and were thereafter compared to the parental control iPSC line. Functional expression of ETO2-GLIS2 upon hematopoietic differentiation of iPSC was validated through quantitative RT-PCR of *ETO2-GLIS2* (Fig. 1B), through ETO2-GLIS2 protein detection (assessed by GFP in the nucleus) and through higher expression of the known fusion target gene *GATA3* in hematopoietic progenitors at day 15 of differentiation, as compared to control iPSC-derived cells (Supplemental Fig. 1E,F, Supplemental Digital Content 1).

**Figure 1:**
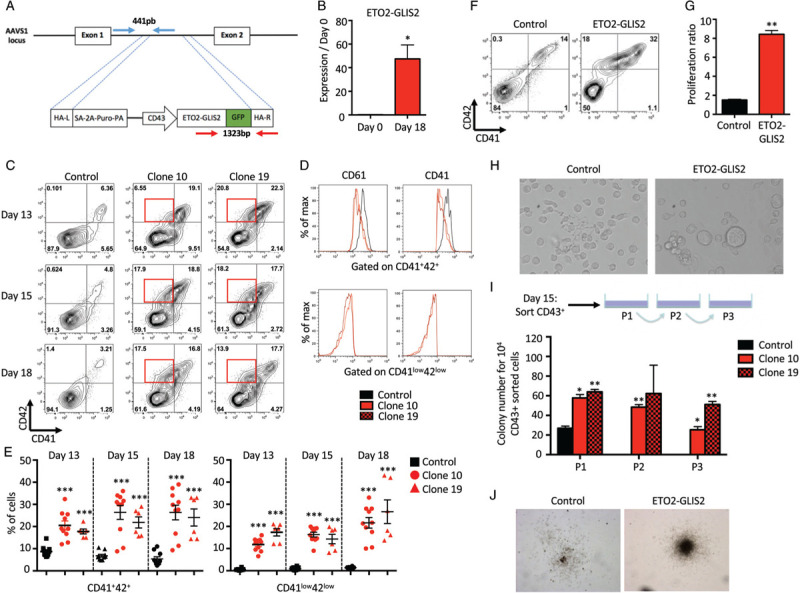
**ETO2-GLIS2 induces self-renewal and differentiation alterations in an iPSC model**. A. Schematic representation of the gene editing strategy. The constitutively active AAVS1 “safe harbor” locus is represented above the targeting construct. The cDNA expression cassette driving expression of *ETO2-GLIS2* cDNA tagged with GFP under the CD43 promoter was inserted by zinc finger-mediated homologous recombination into the AAVS1 intron. HA, homologous arms left (L) and right (R); SA-2APuro-PA, puromycin drug resistance cassette. The position of oligonucleotides used for the genotyping of iPSC clones by PCR are represented by arrows and the expected sizes of the PCR product for the wild-type allele (blue) and the ETO2-GLIS2 recombined (red) locus are indicated. B. Quantitative RT-PCR analysis of *ETO2-GLIS2* expression in iPSC and iPSC-derived day 18 hematopoietic cells from clone 10 relative to *HPRT*. Histograms represent means+/-SD (n = 3 obtained from one differentiation experiment). C. Flow cytometry analysis of CD41 and CD42 megakaryocytic markers in control and clones expressing *ETO2-GLIS2* fusion gene (clone 10 & 19) at day 13, 15 and 18 of differentiation. Red squares highlight the CD41^low^42^low^ population. D. Intensity of the CD41 and CD61 markers gating on the CD41^+^42^+^ populations from control, clone 10 & 19 at day 18 of differentiation (top panel) or on the CD41^low^42^low^ populations from clone 10 & 19 at day 18 of differentiation (bottom panel). E. Percentage of the different populations showed in C. Histograms represent means+/-SD of independent differentiation experiments (Control: n = 10, Clone 10: n = 10, Clone 19: n = 6). F. Flow cytometry analysis of CD41 and CD42 on CD43^+^ hematopoietic cells derived from iPSC at day 15 and further cultured 4 days in liquid culture. G. Proliferation ratio of CD43^+^ hematopoietic cells after 4 days in liquid culture supplemented with Flt3L 10ng/ul, G-CSF 20ng/ul, IL3 10ng/ul, IL6 10ng/ul, SCF 25ng/ul, TPO 10ng/ul and GM-CSF 10ng/ul. Histograms represent means+/-SD (n = 2 obtained from 2 independent differentiation experiments). H. Morphology of CD43^+^ hematopoietic cells after 4 days of liquid culture. I. Upper panel: schematic representation of the experimental design used to test the self-renewal capacity of 10^4^ CD43^+^ sorted cells at day 15 in semi-solid medium. Lower panel: number of colonies as means ± SD (n = 2 obtained from 2 independent differentiation experiments). Cells were replated every 7 days for 4 weeks. P1: first plates; P2: 2^ary^ plates; P3: 3^ary^ plates. J. Representative colonies for control and ETO2-GLIS2 at P1. Statistical significance is indicated as *p*-value (paired Student's *t* test): ^∗∗∗^p < 0.001, ^∗∗^p < 0.01, ^∗^p < 0.05.

Using an updated version of the hematopoietic differentiation protocol of iPSC described by Chou et al^12,13^ (Supplemental Table 2, Supplemental Digital Content 2), we assessed the consequences of ETO2-GLIS2 expression on megakaryocytic differentiation. From control iPSC clones, hematopoietic differentiation is observed by the detection of CD43, CD34, CD41, and CD61 followed by the acquisition of CD42 surface markers (Fig. 1C, 1D and Supplemental Fig. 2A, Supplemental Digital Content 1). CD41^+^CD61^+^CD42^−^ represent progenitors while CD41^+^CD61^+^CD42^+^ represent maturing megakaryocytes. From *ETO2-GLIS2* iPSC clones, a higher fraction of megakaryocytic cells was reproducibly obtained with an increased percentage of CD41^+^CD42^+^ megakaryocytes as early as day13 and sustained at day 15 and day 18 of differentiation (Fig. 1C and E). Of note, *ETO2-GLIS2*^*+*^ iPSC clones maintained a significant production of megakaryocytes over a longer period of time up to day 26, while the number of megakaryocytes generated from control iPSC clones declined after day 22 (data not shown). The *ETO2-GLIS2* iPSC clones also showed an aberrant population characterized by low expression of CD41 and CD42 (named CD41^low^CD42^low^) (Fig. 1C–E). Similarly to CD41, CD61 surface expression was significantly lower in both the *ETO2-GLIS2*-expressing CD41^+^CD42^+^ and, to a higher extent, the CD41^low^CD42^low^ populations, as compared to control cells (Fig. 1D and Supplemental Fig. 2B,C, Supplemental Digital Content 1). These results indicated that *ETO2-GLIS2* expression in human iPSC-derived hematopoietic cells induced differentiation alterations.

We then assessed the proliferation and self-renewal properties of *ETO2-GLIS2*-expressing progenitors. Firstly, we sorted 50,000 CD43^+^ hematopoietic cells from day 15 of differentiation into liquid culture. The output number of cells was counted after 4 days of culture and showed an 8-fold increase in the number of *ETO2-*GLIS2-expressing cells as compared to control cells (Fig. 1F and G). At that stage, morphological studies indicated clear proplatelets formation from controls megakaryocytes while *ETO2-GLIS2*-expressing megakaryocytes were larger and presenting with reduced signs of proplatelets formation (Fig. 1H). Secondly, day15 CD43^+^ hematopoietic cells were sorted into semi-solid medium to perform colony-forming assays scored after 7 to 10 days of culture and weekly serially-replated thereafter (Fig. 1I). While control cells formed colonies only in the first plates, *ETO2-GLIS2*-expressing cells formed larger colonies in the first plates (Fig. 1J) and could consistently form colonies up to the third replating, but not further (Fig. 1I). Importantly, flow cytometry analyses of *ETO2-GLIS2*-expressing cells after replating revealed an important proportion of GPA^+^ and CD43^-^ cells (Supplemental Figure 2D, Supplemental Digital Content 1), supporting the idea of an erythroid differentiation. Of note, injection of day 15 or day 18 CD43^+^ cells from either control or *ETO2-*GLIS2 clones into irradiated immunodeficient NSG mice did not result in significant engraftment with a follow-up of over 9 months (data not shown) indicating that iPSC-derived progenitors obtained using this differentiation protocol are not maintained in vivo in these conditions. Together, these results showed that *ETO2-GLIS2* expression enhances proliferation and self-renewal of iPSC-derived human hematopoietic progenitors in vitro but no long-term in vivo engraftment could be detected in conditioned recipients.

To characterize the molecular consequences of ETO2-GLIS2 expression in iPSC-derived cells, we performed a transcriptome analysis on control and the two ETO2-GLIS2 clones at day18 of differentiation. From the control, the CD41^+^CD42^-^ and CD41^+^CD42^+^ cell populations were sorted to obtain normal immature progenitors and maturing megakaryocytes. From *ETO2-GLIS2-*expressing cells, CD41^+^CD42^+^ and the aberrant CD41^low^CD42^low^ were analyzed (Fig. 2A). As shown by tSNE and PCA plots, replicate conditions and cells from the two clones clustered together and apart from control cells (Fig.2B, Supplemental Fig. 3A, Supplemental Digital Content 1). Gene set enrichment analyses revealed that ETO2-GLIS2^+^ AMKL patient-derived gene expression signatures^7,14^ and ETO2-GLIS2-dependent super enhancer-associated gene signatures^9^ were enriched in ETO2-GLIS2-expressing cells (Fig. 2C, Supplemental Fig. 3B, Supplemental Digital Content 1). Notably, ETO2-GLIS2-expressing CD41^low^CD42^low^ cells showed a significantly higher enrichment of these signatures as compared to ETO2-GLIS2-expressing CD41^+^CD42^+^ (Fig. 2C and D and Supplemental Table 3, Supplemental Digital Content 2) and a significant loss of mature megakaryocyte features assessed by the xCell digital cellular deconvolution method^15^ or by gene ontology associated with a higher expression of *ETO2-GLIS2* (Supplemental Fig. 3C,D,E, Supplemental Digital Content 1). These data indicate that ETO2-GLIS2-expressing CD41^low^CD42^low^ cells present prominent transcriptional alterations that closely recapitulate AMKL patient leukemic cells signature. Expression of ETS and GATA factors are known to control proliferation of megakaryocytes obtained from iPSC^16^ and high ERG and low GATA1 activities are important for ETO2-GLIS2-transformed human blasts.^9^ Therefore, we quantified *ERG* and *GATA1* expression in iPSC-derived hematopoietic cells at different timepoints (day 15, 18, and 22), in AMKL patients or AMKL patients-derived cell lines (eg MO7e) and in a previously described model of ETO2-GLIS2 ectopic expression in HEL cells.^9^*ERG* presented a low basal expression without significant change in ETO2-GLIS2^+^ as compared to control cells (Supplemental Fig. 4A, Supplemental Digital Content 1) and its expression was significantly lower in hematopoietic cells collected from day 22 ETO2-GLIS2^+^ iPSC-derived cells as compared to ETO2-GLIS2-expressing AMKL cells or HEL cells (Fig. 2E). Although GATA1 was moderately repressed by ETO2-GLIS2 at day 15, its expression surprisingly increased in ETO2-GLIS2^+^ cells at day 18 (Supplemental Fig. 4A, Supplemental Digital Content 1) and was significantly higher in day 22 ETO2-GLIS2^+^ iPSC-derived cells compared to ETO2-GLIS2-expressing AMKL cells or HEL cells (Fig. 2E). We then sorted CD41^low^CD42^low^ cells from day 22 ETO2-GLIS2^+^ and showed that they present a higher *ERG* and lower *GATA1* expression compared to ETO2-GLIS2^+^ CD41^+^CD42^+^. However, none of these two populations reached the expression levels observed in the ETO2-GLIS2^+^ MO7e cell line (Supplemental Fig. 4B, Supplemental Digital Content 1). These results demonstrated that ETO2-GLIS2-expressing human iPSC-derived hematopoietic progenitors obtained using this differentiation protocol globally recapitulate the transcriptional program observed in ETO2-GLIS2 AMKL patient leukemic blasts, but lack sustained deregulation of at least the 2 key transcription factors *ERG* and *GATA1*.

**Figure 2:**
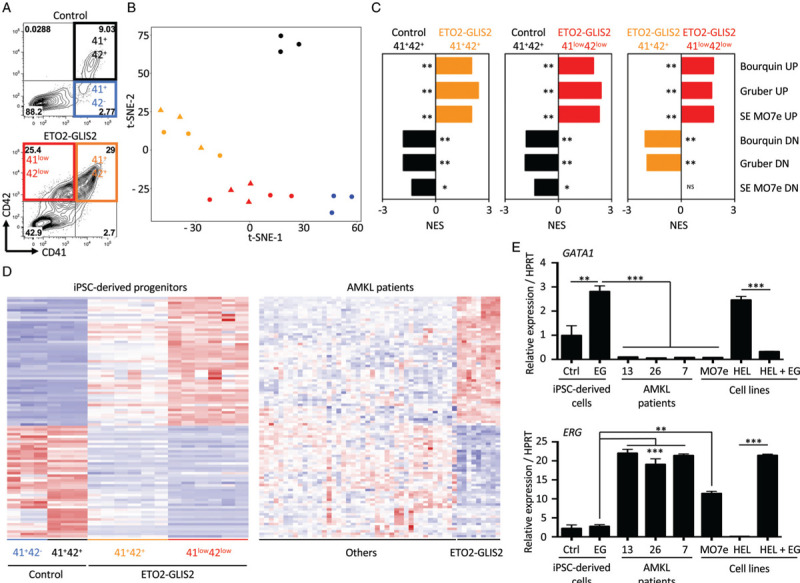
**ETO2-GLIS2 expression in an iPSC model recapitulates molecular features of human AMKL**. A. Flow cytometry plots highlighting the different sorted populations used for RNA sequencing from control and ETO2-GLIS2 (#10 and 19) clones. The color code used throughout the figure is indicated. B. t-SNE clustering of the different populations based on RNAseq data. Blue circles: CTRL CD41^+^CD42^-^, Black circles: CTRL CD41^+^CD42^+^, Orange: ETO2-GLIS2 CD41^+^42^+^ (circles: clone #10, triangles: clone #19), Red: ETO2-GLIS2 CD41^low^42^low^ (circles: clone #10, triangles: clone #19). C. Gene Set Enrichment Analysis comparing indicated paired samples for enrichment in different ETO2-GLIS2-related signatures including signature specific for ETO2-GLIS2^+^ AMKL leukemic cells as compared to other AMKL^7,14^ and ETO2-GLIS2-dependent super enhancer-associated gene signatures (SE). UP: genes upregulated in ETO2-GLIS2^+^ AMKL, DOWN: genes downregulated in ETO2-GLIS2^+^ AMKL. ∗FDR < 0.25, ∗∗ FDR < 0.05. D. Heatmaps of the genes commonly deregulated by the ETO2-GLIS2 fusion in iPSC-derived cells (left) and in AMKL patients leukemic cells (right).^14^ The threshold used to establish the signature was set to fold-change > 2. E. Quantitative RT-PCR quantification of *GATA1* and *ERG* expression in iPSC-derived cells collected at day 22 (Ctrl and EG), in 3 ETO2-GLIS2^+^ AMKL patient leukemic cells (AMKL #13, 26 & 7), in the ETO2-GLIS2^+^ AMKL-patient derived MO7e cell line and in HEL cells (no ETO2-GLIS2 expression) and HEL cells transduced with ETO2-GLIS2 expressing vector.^9^ Histograms represent means+/-SD (n = 3 from independent differentiation experiments for IPSC-derived cells and from technical replicates for AMKL patient samples and cell lines). Ctrl: control; EG: ETO2-GLIS2. Statistical significance is indicated as *p*-value (Student's *t* test): ^∗∗∗^p < 0.001 ^∗∗^p < 0.01.

Here, we report the first model of ETO2-GLIS2 expression in human hematopoietic progenitors obtained from the precise gene editing of healthy iPSC. Our data demonstrate that ETO2-GLIS2 impairs normal megakaryocytic differentiation, increases self-renewal and globally recapitulates the transcriptional program observed in human AMKL leukemic blasts. Importantly, we identified in ETO2-GLIS2^+^ iPSC-derived hematopoietic cells relevant molecular differences associated with their differences in self-renewal and long-term growth properties as compared to human AMKL patient leukemic blasts. Indeed, *ERG* expression is not strongly induced in ETO2-GLIS2^+^ iPSC-derived progenitors and remains at a low level compared to its expression in ETO2-GLIS2 AMKL patient leukemic cells. In addition, the lack of sustained repression of *GATA1* is consistent with the capacity of these cells to undergo erythroid differentiation upon serial replating and suggest that ETO2-GLIS2 does not fully block erythroid differentiation in these conditions. Therefore, we propose that the transient increase in self-renewal and altered differentiation in this human iPSC model of ETO2-GLIS2 expression results, in part, from an insufficient deregulation of the ERG/GATA1 activities. Of note, these characteristics could be independent of ETO2-GLIS2 and may result from the type of hematopoiesis obtained with in vitro differentiation of iPSC to date. Indeed, it is generally accepted that current in vitro differentiation protocols do not reach an adult definitive hematopoiesis and it is still debated whether they recapitulate a primitive or transient definitive (EMP-like) hematopoiesis.^17^ Whether the strong expression of CD43 in the primitive streak^18^ may explain the results obtained here and would warrant the use of a different strategy to control ETO2-GLIS2 expression or whether a different differentiation protocol will be required to obtained definite hematopoiesis and full-blown transformation should be interesting areas of investigation. Together, this modeling approach in iPSC represents an important tool to understand the cellular and molecular bases for the transition between normal to malignant hematopoiesis, to develop more faithful models of childhood leukemia using human cells and to perform preclinical drug testing for the development of novel therapeutic strategies.

## Acknowledgments

We are grateful to Mitchell Weiss for providing the AAVS targeting vectors, to Olivier Bernard and Eric Solary for facilitating this collaborative effort, to Esteve Noguera and Daniel Gautheret for bioinformatics support and Paule Zanardo for excellent administrative assistance.

## Supplementary Material

Supplemental Digital Content

## Supplementary Material

Supplemental Digital Content
