# Supplementary material for: The Pediatric Acute Leukemia Fusion Oncogene ETO2-GLIS2 Increases Self-Renewal and Alters Differentiation in a Human Induced Pluripotent Stem Cells-Derived Model
Source: Hemasphere. 2019 Dec 16;4(1):e319. doi: 10.1097/HS9.0000000000000319 (PMC7000481; doi:10.1097/HS9.0000000000000319)
Supplement: Supplemental Digital Content [file hs9-4-e319-s001.pdf]

SUPPLEMENTAL INFORMATION

-Supplemental Figure & Table legends

-Methods

SUPPLEMENTAL FIGURES & TABLE LEGENDS

Supplemental Figure 1

A- PCR screening for the endogenous or ETO2-GLIS2-targeted AAVS1 locus. The expected sizes for the PCR products obtained from amplification of the wild-type allele and the ETO2-GLIS2 recombined allele (represented Figure 1A) are indicated.

B- Karyotype for control and #10 iPSC clones.

C- Quantitative RT-PCR on RNA from embryoid body (EB) formed after 24 days of differentiation. Expression is represented as a ratio between day 24 EBs and undifferentiated iPSC cells. Genes characteristic of the undifferentiated state (*NANOG*, *OCT4*) and of the three embryonic layers, mesoderm (*GATA4*), endoderm (*HAND1*) and ectoderm (*NeuroD1*) were used.

D- Histological section of teratoma formed after injection of iPSC into NSG mice.

E- Confocal fluorescence microscopy images obtained at day 15 of differentiation (left panel: control, right panel: ETO2-GLIS2).

F- Quantitative RT-PCR at day 15 of differentiation for *GATA3* expression. Histograms represent means+/-SD (n=3 obtained from one differentiation experiment). Statistical significance is indicated as p values (Student's t-test): **p-value<0.01

Supplemental Figure 2

A- Flow cytometry analysis of CD43 and CD34 hematopoietic markers in control and clones expressing *ETO2-GLIS2* fusion gene (clone 10 & 19) at day 13, 15 and 18 of differentiation.

B- Mean fluorescent intensity of CD41 and CD61 surface markers in the indicated populations. Mean+/-SD (n=3 independent differentiation experiments) are represented. Statistical significance is indicated as *p*-value (Student's t-test): ***p<0.001 **p<0.01.

C- Intensity of the CD42 marker gating on the $CD41^{+}42^{+}$ populations from control, clone 10 & 19 at day 18 of differentiation (up-left panel) or on the $CD41^{low}42^{low}$ populations from clone 10 & 19 at day 18 of differentiation (up-right panel). Mean fluorescent intensity in the indicated populations. Mean+/-SD (n=3 independent differentiation experiments) are represented. Statistical significance is indicated as *p*-value (Student's t-test): ***p<0.001 **p<0.01.

D- Flow cytometry analysis of CD43 and GPA markers for control and ETO2-GLIS2 cells at different stages of methylcellulose culture serial replating. P1: first plates; P2: 2^{ary} plates; P3: 3^{ary} plates.

Supplemental Figure 3

A- Principal component analysis of the samples used for RNAseq. Color code is the same than in Figure 2. Blue: CTRL $CD41^{+}CD42^{-}$, Black: CTRL $CD41^{+}CD42^{+}$, Orange: ETO2-GLIS2 $CD41^{+}42^{+}$, Red: ETO2-GLIS2 $CD41^{low}42^{low}$.

B- Gene Set Enrichment Analysis plots comparing ETO2-GLIS2 $CD41^{low}42^{low}$ versus ETO2-GLIS2 $CD41^{+}42^{+}$ comparison with the indicated lists at the top of each panel. False discovery rate (FDR) and Normalized Enrichment Score (NES) are indicated.

C- ETO2-GLIS2 expression in the different populations for control ($CD41^{+}42^{-}$ & $CD41^{+}42^{+}$) and ETO2-GLIS2 clones ($CD41^{+}42^{+}$ & $CD41^{low}42^{low}$) from RNAseq. Histograms represent means+/-SD (n=3 independent differentiation experiments).

D- Top 10 Gene Ontology (GO) biological process categories enriched in the up- or down-regulated signatures of the ETO2-GLIS2 $CD41^{low}42^{low}$ population as compared to control and ETO2-GLIS2 $CD41^{+}42^{+}$ cells. The scale represents the combined score calculated based on the z-score enrichment and the p-value as indicated by the Enrichr online software.

E- Digital cellular heterogeneity portrait of each transcriptome using the xCell method (14).

Supplemental Figure 4

A- Quantitative RT-PCR expression of *ERG* and *GATA1* in hematopoietic cells obtained at day 15, 18 and 22 from control and ETO2-GLIS2 #10 iPSC clone normalized to *HPRT*. Histograms represent means+/-SD (n=3 obtained from one differentiation experiment).

B- Quantitative RT-PCR expression of *ERG* and *GATA1* normalized to *HPRT* in purified hematopoietic cells obtained at day 22 from control iPSC ($CD41^{+}42^{+}$ population) and ETO2-GLIS2 iPSC clones 10 and 19 ($CD41^{+}42^{+}$ and $CD41^{low}42^{low}$ populations) as compared to HEL (no ETO2-GLIS2 expression) and MO7e (ETO2-GLIS2+ patient-derived) cell lines. Histograms represent means+/-SD (duplicate from two independent experiments).

Statistical significance is indicated as *p*-value (Student's t-test): ***p<0.001 **p<0.01. *p<0.05.

Supplemental Table 1

List of primers used in this study.

Supplemental Table 2

Growth factors used for the hematopoietic differentiation of iPSC.

Supplemental Table 3

Table of counts (RPKM) for the genes deregulated in common in both iPSC and AMKL leukemic blasts.

METHODS

Cloning of the *ETO2-GLIS2* targeting construct

ETO2-GLIS2-GFP was cloned under the control of the human CD43 promoter into the AAVS1-SA-2A-puro-pA a kind gift of M. Weiss (St. Jude Children's Research Hospital, Memphis, TN, USA) through in-fusion strategy following manufacturer's instructions (In-Fusion® HD Cloning Kit, Takara Bio, USA) and transformation into XL10-Gold Ultracompetent cells (Agilent). The other two vectors encoding the two Zing Finger proteins specific for the AAVS1 locus (left and right arms) were also obtained from M. Weiss.

iPSC culture and editing

iPSC were derived from healthy donor CD34+ cells as described previously (11). iPSC were cultured in Essential 8 medium (Thermofisher) and in vitronectin (Thermofisher) coated wells. iPSC were passed every 3-4 days in DPBS/0.5mM EDTA Ultrapure (Thermofisher). A Mycoplasma screening was routinely performed, according to the manufacturer instructions (Sigma, Saint-Quentin Fallavier, France).

To obtain iPSC presenting pCD43-*ETO2-GLIS2-GFP* integration at the AAVS1 locus, wild-type iPSC were treated with Triple 1x (Thermofisher) to make single cells and $0.8x10^{6}$ single cells were electroporated with the *ETO2-GLIS2* donor construct in association with the vectors encoding for Zing Finger, using Nucleofector 2B and Stem cell kit (Lonza). Electoroporated iPSC were plated on fresh vitronectin-coated plates in presence of 10µM of Y27632 (Miltenyi Biotec). Three days later the electroporated iPSC were selected with puromycin (0.5 µg/mL) for 48h. After 1 week, 23 individuals clones were picked, expanded individually and screened by PCR for correct integration at the AAVS1 locus. Two pairs of oligonucleotides were used to detect the correct integration. One was used for the recombined allele (expected PCR product size: 1323 bp) and another one was used for the endogenous allele (expected PCR product size: 441bp). Homozygous clones were defined as positive for the recombined allele PCR product and negative for the endogenous allele PCR product.

Characterization of iPSC with *ETO2-GLIS2* Knock-in

Wild-type iPSC and iPSC from clone 10 with ETO2-GLIS2 knock-in were dissociated in single cells through Triple 1x treatment. $1x10^{6}$ cells were plated in Nunclon™ Sphera (ThermoFisher) to induce embryoid bodies development. Embryoid bodies (EB) were grown in Essential 6 Medium (ThermoFisher) replacing medium every 3 days. RNA from embryoid bodies were extracted after 24 days and the expression of markers from the three layers upon EB differentiation were tested for iPSC CTRL and iPSC clone 10. The list of all primers used for PCR and RT-PCR is reported in Supplementary Table 1.

Hematopoietic differentiation of iPSC

Three clones with ETO2-GLIS2 and CTRL iPSC were induced to differentiate into hematological lineage using 2D co-culture system on matrix adapted from (12). Briefly 3-4 colonies of each iPSC were picked and put into Geltrex (Thermofisher) coated wells in Essential 8 medium. For the first 2 days, the cells are grown in StemPro34 (SP34) medium supplemented with 1X penicillin/streptomycin (PS, GIBCO), glutamine (2 mM), ascorbic acid (50 µg/ml, Sigma) monothioglycerol (MTG, 15 mM, Sigma), BMP4 (5 ng/mL) and VEGF (50 ng/ml) and CHIR (930 ng/mL). The medium was replenished and supplemented until day 18 with a sequential cytokine cocktail, as shown in Table 2. Hematopoietic differentiation of iPSC was then assessed at day 13, 15 and 18 using flow cytometry analyses for hematopoietic progenitors and megakaryocytic markers. All flow cytometry antibodies were purchased from BD Biosciences (CD34-PECy7: #560710, CD43-APC: #560198, CD41-V450: #561425, CD42-PE: #561854, CD61-APC: #564174, CD235a-PE: #561051). To test the proliferation properties of hematopoietic progenitors, CD43+ sorted cells were cultured in SP34 medium supplemented with Flt3L 10ng/ul, G-CSF 20ng/ul, IL3 10ng/ul, IL6 10ng/ul, SCF 25ng/ul, TPO 10ng/ul and GM-CSF 10ng/ul for 4 days. All data were analyzed with FACSCantoII or Fortessa with FACS Diva software (BD Biosciences).

Methylcellulose culture

To test self-renewal properties CD43+ hematopoietic cells derived from iPSC differentiation were sorted by flow cytometry and cultured in methylcellulose (H4230, StemCell Technologies) enriched with Flt3L 10ng/ml, G-CSF 20ng/ml, IL3 10ng/ml, IL6 10ng/ml, SCF 25ng/ml, TPO 10ng/ml, GM-CSF 10ng/ml. All cytokines used were purchased from Peprotech, UK. Cells were replated every 7 days for 4 weeks.

DNA/RNA extraction and RT-PCR

DNA and RNA were isolated using the RNeasy Mini and All prep DNA/RNA Micro Kit (Qiagen) and quantified by NanoDrop (ThermoScientific). Reverse transcription was done with SuperScript II from Invitrogen. Quantitative-PCR for ETO2-GLIS2 was performed using SYBR Select master mix (Applied Biosystems) on 7500HT Fast Real-Time PCR System (Applied Biosystems) following manufacturer's recommendations. All Primers used were reported in Supplementary Table 3. *GATA3* and *ERG* expression was tested with TaqMan probe (Applied Biosystem). *GATA3*: Hs00231122, *ERG*: Hs01554629.

Transcriptome sequencing & bioinformatics analyses

Whole transcriptome sequencing (WTS) was performed at the "Giorgio Prodi" Cancer Research Center using Illumina Nextseq500 platform. cDNA libraries were synthesized from 250 ng total RNA using the TruSeq Stranded mRNA kit (Illumina) following manufacturers' instructions. Briefly, poly-A

containing mRNA molecules were reverse transcribed to double stranded cDNA fragments that were then adenylated at 3' ends and ligated to single-index adapters. PCR-enriched libraries were then quantified by Quant-It picogreen assay (Thermo-Fisher) and sized with the High Sensitivity kit on the 2100 Bioanalyzer (Agilent Technologies). Sequencing was performed at 2x80bp using Illumina Sequencing by synthesis (SBS) technology, yielding an average of 45.68 million reads per sample.

Analysis were performed on R (3.5.1 version) using heatmap3 (1.1.6), Rtsne (0.15) and ggplot2 (3.1.1) packages. Gene set Enrichment Analysis were perform using GSEA software from the Broad Institute. Lineages enrichment analyses were perform using the xCell software (14). Gene Ontology was performed using online gene ontology resource (http://geneontology.org)

Statistical analyses

Statistical analyses were performed using the GraphPad Prism software (version Prism 6-2) except otherwise mentioned.

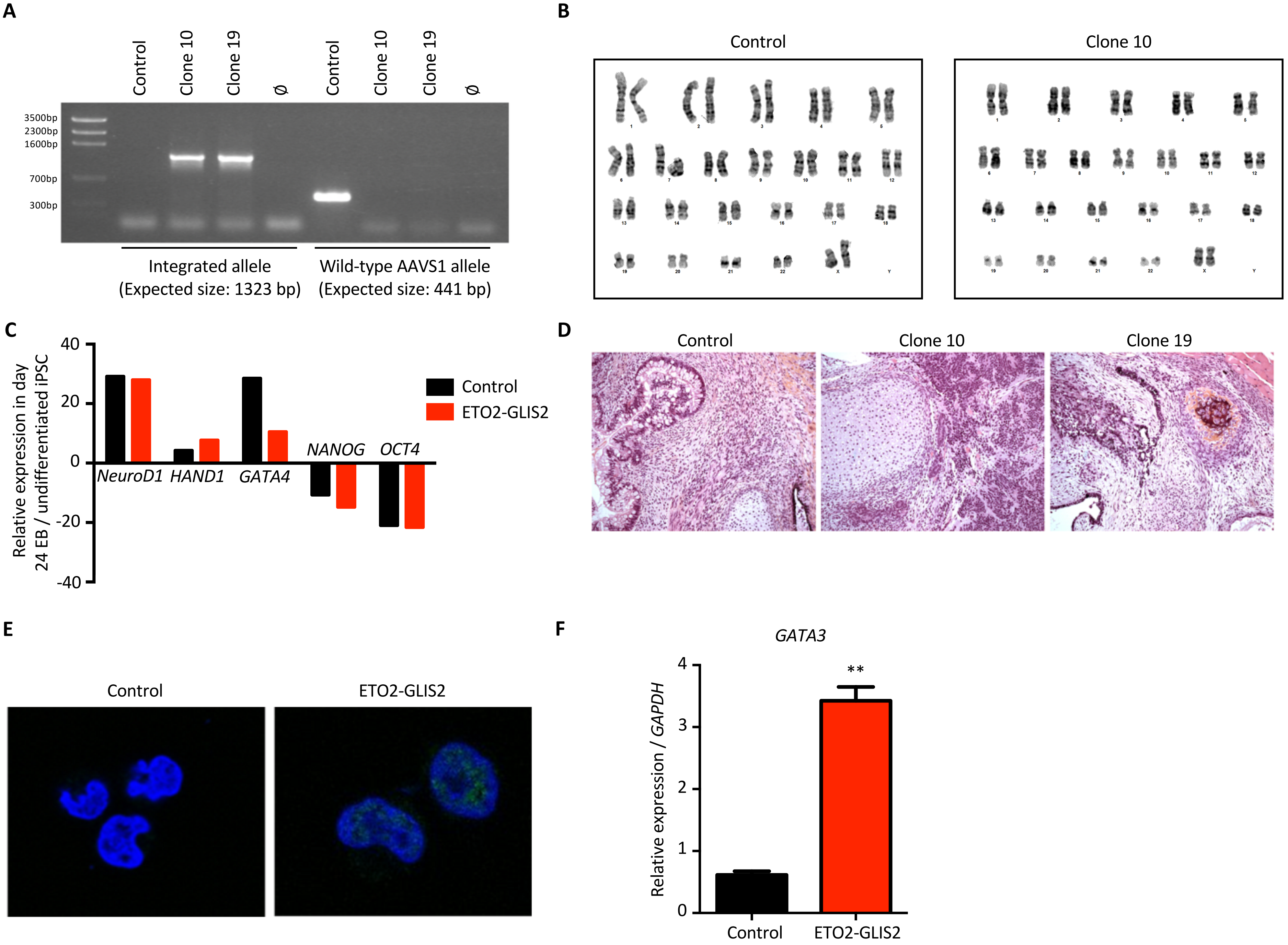

Supplementary Figure 1

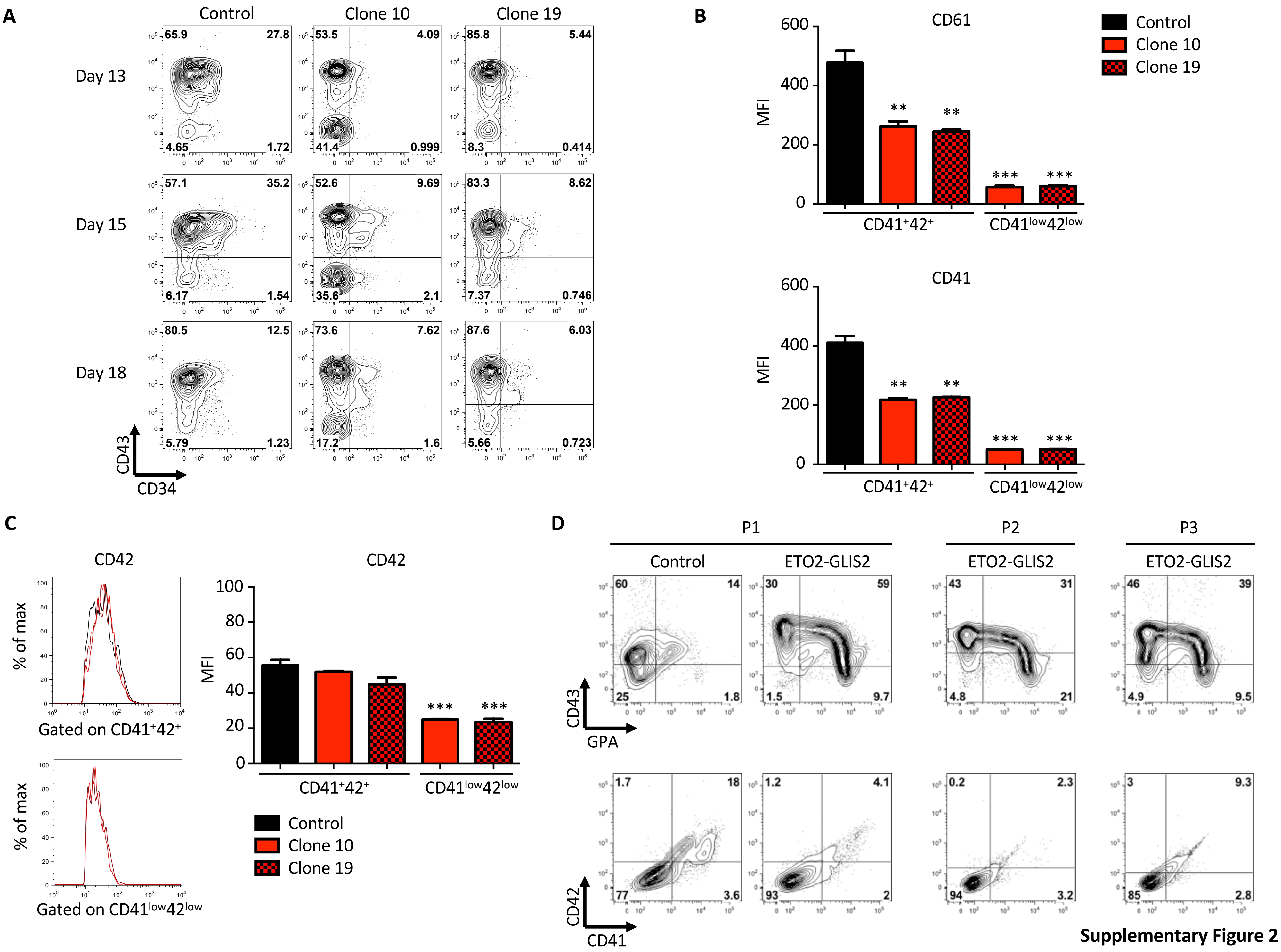

Supplementary Figure 2

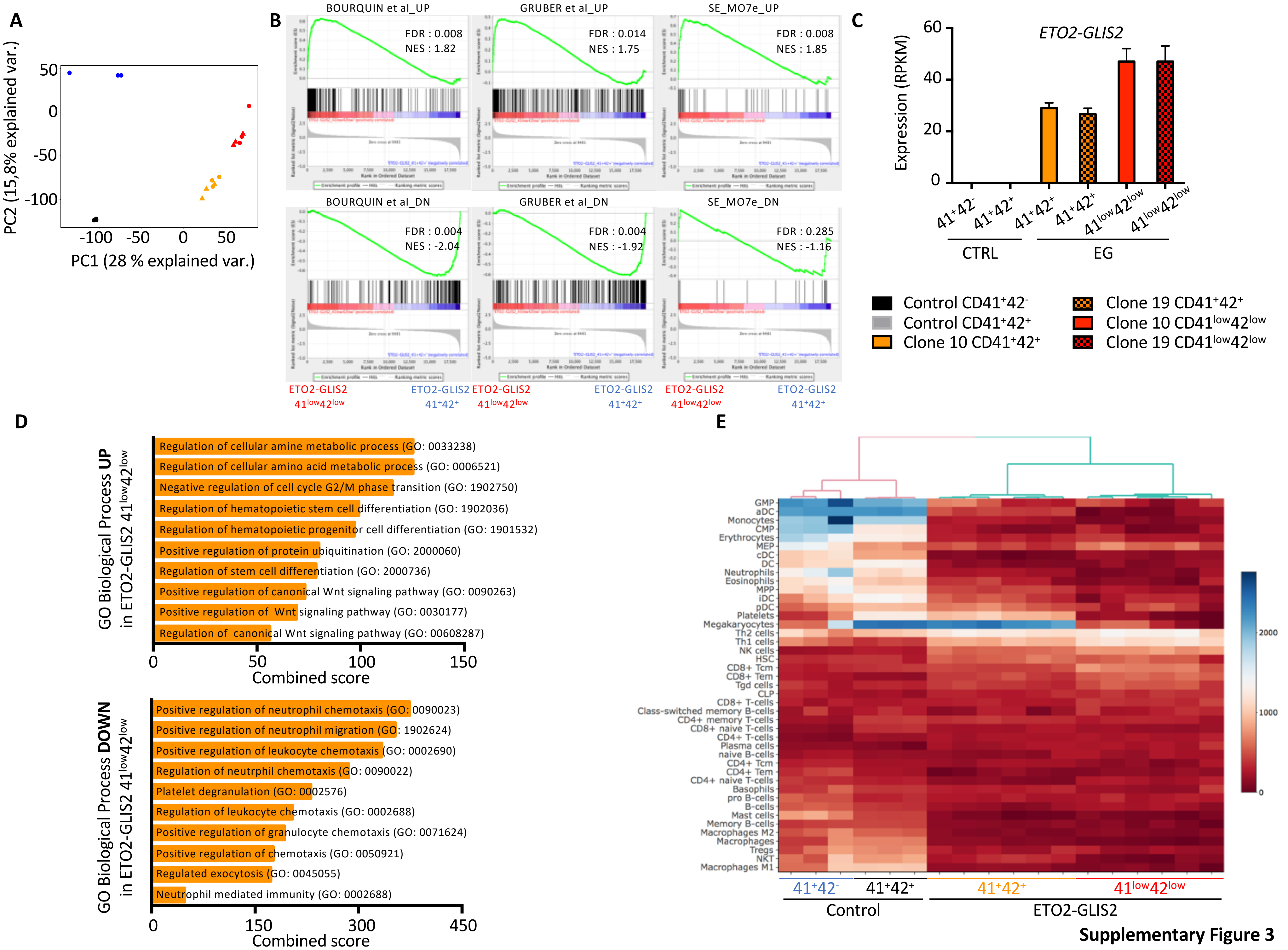

Supplementary Figure 3

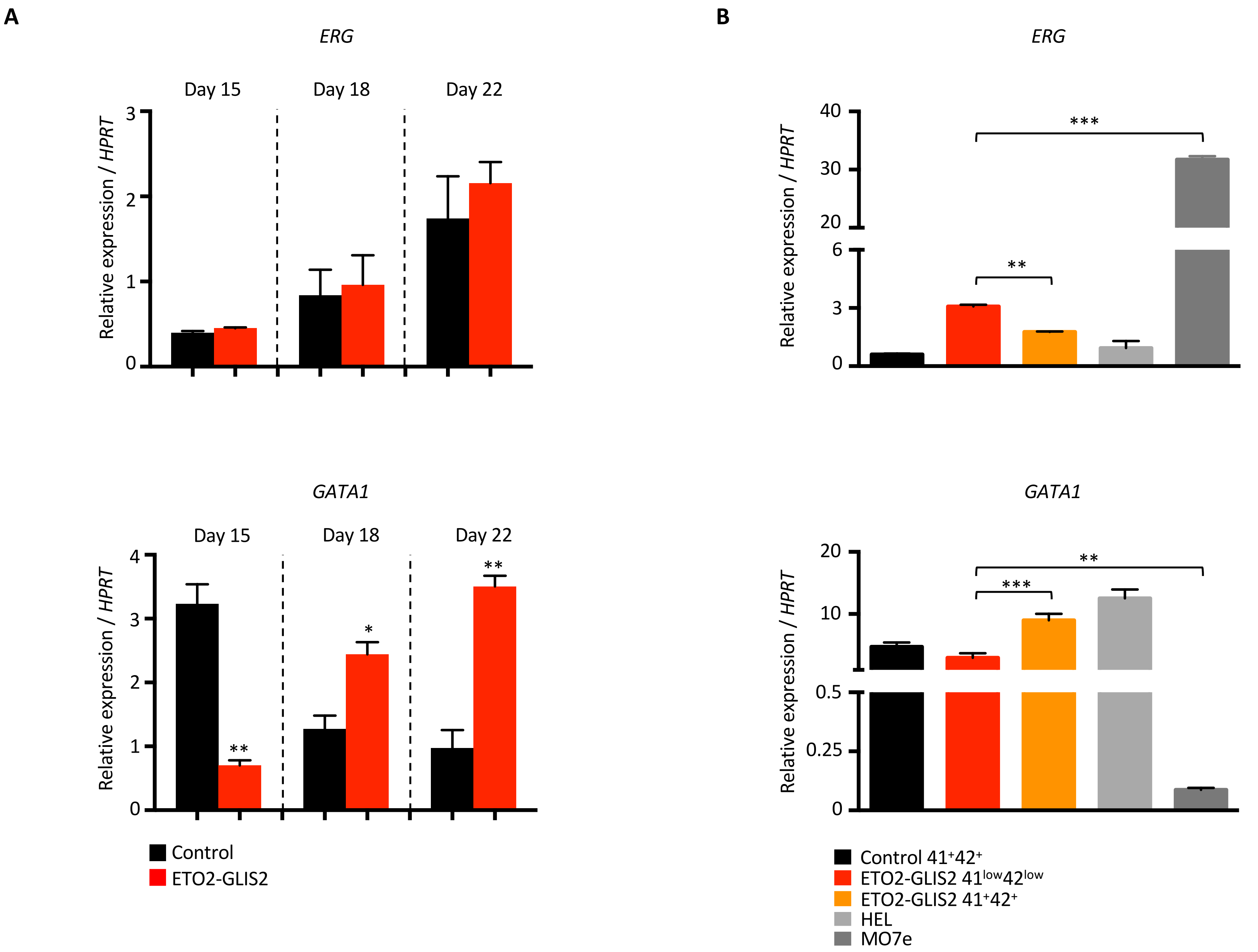

Supplementary Figure 4
